# Breathing modes of Kolumbo submarine volcano (Santorini, Greece)

**DOI:** 10.1038/srep46515

**Published:** 2017-04-13

**Authors:** Evangelos Bakalis, Theo J. Mertzimekis, Paraskevi Nomikou, Francesco Zerbetto

**Affiliations:** 1 Dipartimento di Chimica “G. Ciamician”, Universita di Bologna, Italy; 2 Department of Physics, National and Kapodistrian University of Athens, Zografou Campus, GR-15784, Athens, Greece; 3 Department of Geology and Geoenvironment, National and Kapodistrian University of Athens, Zografou Campus, GR-15784, Athens, Greece

## Abstract

Submarine volcanoes, such as Kolumbo (Santorini, Greece) are natural laboratories for fostering multidisciplinary studies. Their investigation requires the most innovative marine technology together with advanced data analysis. Conductivity and temperature of seawater were recorded directly above Kolumbo’s hydrothermal vent system. The respective time series have been analyzed in terms of non–equilibrium techniques. The energy dissipation of the volcanic activity is monitored by the temperature variations of seawater. The venting dynamics of chemical products is monitored by water conductivity. The analysis of the time series in terms of stochastic processes delivers scaling exponents with turning points between consecutive regimes for both conductivity and temperature. Changes of conductivity are shown to behave as a universal multifractal and their variance is subdiffusive as the scaling exponents indicate. Temperature is constant over volcanic rest periods and a universal multifractal behavior describes its changes in line with a subdiffusive character otherwise. The universal multifractal description illustrates the presence of non–conservative conductivity and temperature fields showing that the system never retains a real equilibrium state. The existence of a repeated pattern of the combined effect of both seawater and volcanic activity is predicted. The findings can shed light on the dynamics of chemical products emitted from the vents and point to the presence of underlying mechanisms that govern potentially hazardous, underwater volcanic environments.

Kolumbo is a shallow submarine volcano, part of the Hellenic Volcanic Arc, located 7 km NE of Santorini^1^,^2^,^3^, and is considered as one of the most active and dangerous submarine volcano in the Mediterranean^4^. In 2006, an active hydrothermal vent field (HVF) was discovered on the north part of Kolumbo’s crater, at 504 m below sea level^1^, comprising polymetallic (Au, As, Sb, Hg, Ag, Tl, Zn, Pb) active and inactive sulfide–sulfate hydrothermal chimneys and mounds^5^. The *in situ* study of hydrothermal vent activity can provide important information on the detailed properties of the volcanic system, especially on the dynamical processes taking place. In that aspect, an exploration campaign in 2010 and 2011 focused –among other things– on the intense vent activity and the continuous exchange of gases and hydrothermal fluids. In the water column, important anomalies in temperature and conductivity depth profiles were also recorded^5^,^6^,^7^,^8^. Detailed investigation of the vent activity of a submarine volcano is usually performed in terms of the study of the ^3^He/^4^He ratio, as this is one of the most powerful geochemical tracers to define the origin of volatiles released from the solid earth and the magmatic/mantle features. The mantle below Kolumbo has a ^3^He/^4^He signature of at least 7.0 Ra suggesting that this submarine volcano is characterized by a vigorous activity with a potential volcanic hazard^8^.

Exploration of the Kolumbo shallow submarine volcano took place aboard E/V “*Nautilus*” using the state–of–the–art Remotely Operating Vehicles (ROV) “*Hercules*” and “*Argus*”, in the summers of 2010 and 2011. In 2010, the NA007 research cruise was dedicated on the first–ever detailed study of Kolumbo’s crater (dates: 29 July–12 August 2010)^9^, while in 2011 further investigation of the HVF was one of the main objectives of research cruise NA014 (dates: 01–10 September 2011)^10^. Conductivity–Temperature–Depth (CTD) data used in the present study were recorded *in situ*, just above the HVF in the north part of the seafloor inside Kolumbo’s crater, at a maximum depth of 504 m below sea level. The CTD data were recorded using a ROV–mounted sensitive Seabird CTD probe of 16 Hz sampling rate, while the submersible ROV was hovering the HVF performing simultaneously several other types of measurements (see more details published elsewhere)^6^,^7^. Such CTD measurements differ significantly from those recorded by stationary instruments which record long time series and may detect seasonal variations. The analyzed CTD data correspond to a time interval of a full hour when the ROV stayed directly above the HVF sources: in 2010 the activity was particularly intense, while in 2011 the activity vanished almost completely (Fig. 1). These observations are in complete agreement with other sets of data and visual observations^2^,^5^,^8^.

Besides the study of geochemical tracers, additional properties can shed light on the mechanisms underlying the volcanic activity. In the present work, for the first time to the best of our knowledge, the effect of the presence of a submarine volcano on two seawater properties, i.e. temperature, Θ, and conductivity, *E*_*c*_, is analyzed. Both Θ and *E*_*c*_ reflect on the way the system functions, namely variations in temperature reflect the energy dissipation, while conductivity changes monitor the dynamics of venting chemical products (ions, gases). For both properties examined here, *in situ* recorded data have been used.

The time series depicted in Fig. 1 may be analyzed by assuming that they are manifestations of stochastic processes. Traditionally, the classification of a random process, *x(t*), is made on the way variance, *W(t*) = 〈*x*^2^(*t*)〉 − 〈*x(t*)〉^2^, scales with time. For a pure random process the variance is proportional to the elapsed time, 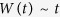, while for a random process deviating from the norm, the variance reads^11^


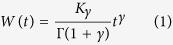


where *K*_*γ*_ is the generalized coefficient expressed in proper units, e.g. if temperature is the property under study then the units are deg^2^s^−*γ*^, Γ() is the Gamma function, and *γ* is the exponent that classifies the type of the process: normal or Brownian for *γ* = 1, sub–normal or subdiffusive for 0 < *γ* < 1, and supernormal or superdiffusive for 1 < *γ* < 2. For discrete data sets, equation (1) takes the form^12^,^13^


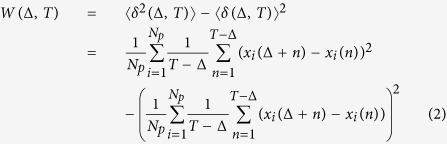


where *N*_*p*_ is the number of realizations and for *N*_*p*_ = 1 the first summation is omitted. In application of equation (2), we run a time sliding moving average covering all possible combinations that include data points with the same elapsed time. The total length of the trajectory, *T* = *N* × *τ*, is expressed in terms of the elapsed time between two consecutive measurements, *τ*, or minimum lag time, which is the reciprocal of the sampling rate, *f*. The lag time, Δ, in equation (2), takes the role of the time in equation (1), and its values are in the range, *τ* ≤ Δ ≤ *T*/10, in order to have reliable statistics.

The variance in either continuous, equation (1), or discrete space, equation (2), is expressed in terms of the first two moments of the probability density function *pdf*. This suffices when we deal with random processes drawing steps from a Gaussian distribution. Nevertheless, if extreme events contribute significantly to the *pdf*, which can no longer be described by a Gaussian distribution, more moments of the *pdf* are necessary to be obtained.

The method of *generalized moments* (GMM)^14^,^15^,^16^,^17^, see Equation (3), takes into account moments lower and higher than two, where moments lower than two describe the core of the *pdf*, while moments higher than two describe the tail of the *pdf*. GMM has been applied successfully in diverse fields^12^,^13^,^18^,^19^,^20^. The GMM approach uses the moments of the absolute value of the change between two consecutive steps, and it works as follows. Assuming *x*_*i*_ is a random process with *i* = 1, …, *N (N* is the total number of data points or steps), and the sampling frequency, *f*, is constant then *τ* = 1/*f* is the smallest lag time and *T* = *N* × *τ* is the total time. Depending on the choice of the lag time, Δ, we can construct a number of time series, which contain the absolute change between two data points of the initial series, separated by the lag time, 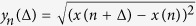, with *τ* ≤ Δ ≤ *T*/10. Then the moments of *y*_*n*_(Δ), 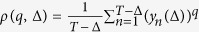 can be obtained. If *q* stands for the order of the moment and Δ is the lag time then according to this method the moments *ρ(q*, Δ) are expected to scale with Δ as


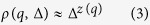


where *z(q*) is the structure function which identifies, when possible, the stochastic mechanism, or mechanisms, that drive the random process. Furthermore, the shape of *z(q*) classifies the process either as mono– or as multifractal, i.e. monofractal process for linear form, *z(q*) = *hq*, and multifractal for any convex shape^20^. Among multifractals, universal multifractals are likely to be ubiquitous^21^,^22^,^23^. For universal multifractals the structure function of equation (3) takes the form^23^:


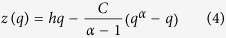


If Φ is a field (property) that is studied through the analysis of the time series then the variables *h, C*, and *α* inserted in equation (4), have the following meaning: *h* = *z*(1) defines the scaling of the mean field of Φ, and for *h* = 0, Φ is a conservative field. *C*, with *C* > 0, measures the mean homogeneity of the field of Φ, the larger *C* the more the mean field is inhomogeneous or fractal. *α*, with 0 ≤ *α* *<* 2 is the Lévy index, that is, the *α*–stable L*é*vy distribution from which the process draws its changes^24^. The value of *α* shows how fast the inhomogeneity increases with the order of the moments. For *α* = 1, *z(q*) = *hq* − *Cq*log(*q*), and the distribution draws changes from a Cauchy–Lorentz distribution.

## Results

In applying the GMM method to *E*_*c*_ and Θ time–series data, different moments up to the 4th order have been constructed (Fig. 2). The moments for the 2011 Θ data are parallel to the time axis (Fig. 2d). On the other hand, 2011 *E*_*c*_ data show dependence on the different lag times. A unique fit for each moment, see (Fig. 2c), and for all lag times captures well the dynamics exclusively for large times. Instead, there is a crossing point at Δ = 1 s (vertical solid line in the graph), where the fits work well simultaneously for small and large lag times. This can be interpreted by assuming that the respective dynamics are different. The moments for 2010 show a crossing point at Δ = 5 s in the underlying dynamics for both *E*_*c*_ and Θ (vertical solid line in both graphs). For every moment, data in two different regimes have been fitted with the equation *f*(Δ) = *c*Δ^*b*^. For 2010 data, Δ ≤ 5 for the first regime, and 5 ≤ Δ ≤ 150 for the second regime. For 2011 data, Δ ≤ 1 for the first regime, and 1 ≤ Δ ≤ 100 for the second regime. It should be noticed that the variance shown in Fig. 4 has an additional third regime, which corresponds to Δ ≥ 150 and Δ ≥ 100, respectively for 2010 and 2011 data sets. The exponent *b* corresponds to the value of the structure function, *z(q*), for a specific value of moment *q* in equation (3).

**Figure 1:**
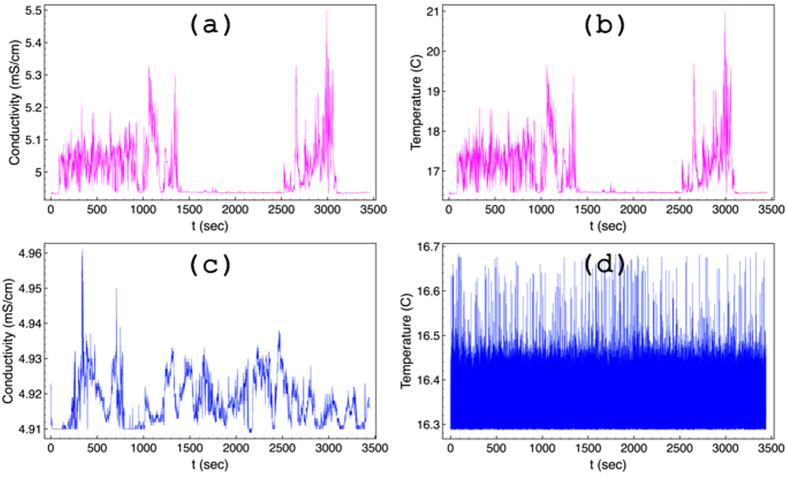
Representative time series data for 2010 *E*_*c*_ (**a**) and Θ (**b**) when vent activity was intense, and 2011 *E*_*c*_ (**c**) and Θ (**d**) when the hydrothermal vent field was inactive. Please note the largely different scales of y–axes in (**a**–**d**).

The structure functions are illustrated in Fig. 3. The convex shape of three out of four structure functions implies that the time series in Fig. 1 corresponds to multifractal processes. All structure functions, with the exception of the first regime of *E*_*c*_ in 2011, satisfy equation (4). The exception fits nicely to equation (4) with an additive logarithmic term, *z(q*) − *d*log(*q*), where the correction term could possibly be attributed to the limited number of data points in this regime or, perhaps, to underlying physics, as in the case of the 2D Ising model^25^,^26^. All the estimated parameters of the structure function obtained by fitting with equation (4) are listed in Table 1.

For 2010 *E*_*c*_ data in the first regime, which covers data points with a maximum lag of 5 s, the mean field of *E*_*c*_ is inhomogeneous, *C* is equal to 0.133, and the Cauchy–Lorentz, *α* = 1, distribution describes its changes. The scaling factor, *h* = 0.528, refers to a non–conservative conductivity mean field. The latter underlines the presence of a magnetic field, which is the result of the motion of the outgoing ions from the crust to the water through the underwater volcanic chimneys. The same value of *h* would indicate superdiffusion if the process were monofractal; however, the overall process is subdiffusive with exponent 0.874 (see Fig. 4).

As the window of lag times increases, 5 ≤ Δ < 150 s, the conductivity mean field becomes almost homogeneous, *C* = 0.03, its changes are drawn by a 1.619–stable Lévy distribution, and the *E*_*c*_ field remains non–conservative, *h* = 0.185. The overall process is strongly subdiffusive, with the characteristic exponent equal to 0.315. In the third regime of lag times (see Fig. 4), for Δ ≥ 150 s, where the structure function is not shown in Fig. 3, the variance is almost constant, the exponent is 0.038, assuring that the conductivity is practically unchanged. The overall picture is that raw data close to each other share a memory; the motion is anti–persistent, implying a temporal increase of the conductivity is probably followed by a step where conductivity decreases. This memory kernel becomes stronger in the second regime, and leads to a complete trapping in the last regime. Notice that the term trapping herein is used to point unchanged properties. Since measurements were collected at the same location, the difference between lag times indicates ions are produced by volcanic activity at different time moments. The strong subdiffusive behavior of the second regime, and most importantly the trapping in the third one, underlines that outgoing products, with large difference of lag time, share common properties.

Pictorially, one could say that ions, which are direct products of the volcanic vent activity, leave the crater in a manner that resembles the breathing in time of the volcano. Each breath sends out a mixture of products and is probably repeated after a period, forming the conductivity changes as discussed above. The combined effect of seawater and volcanic activity retains a memory, see Fig. 5b. This is in complete agreement with both visual observations of vent outbursts in form of 99%-rich-in-CO_2_ bubbles^6^,^8^ and observations of outflowing, sulphur–rich, diffusers.

This observation is further examined by means of the behavior of the normalized velocity autocorrelation function (NVAF), as well as the behavior of the memory kernel function, *k(t*)^12^,^27^ (Fig. 5). For each lag time, Δ, the form of the NVAF confirms the subdiffusive character of the process. The NVAF becomes negative, thus indicating that every new step of the process is likely opposite to the previous one, highlighting its anticorrelated nature. The memory retained by the process is estimated via *k(t*), which is connected to NVAF through an integrodifferential equation. For discrete data sets this equation can be solved recursively^27^. For the lag time of 0.06 s, *k(t*) is symmetric and oscillates around zero indicating that conductivity measurements separated by the minimum lag time do not likely share any common memory. This is in line with the scaling exponent of this regime, 0.874, close to 1, which stands for a pure random process. Increasing the lag time to 5.1 s, *k(t*) becomes positive for the first 5 s and then turns to oscillate around zero. This means that a memory being formed for sampling data separated by 5 s. The memory kernel presents the same behavior for lag time of 150 s, which is quite asymmetric for the first 150 s taking positive values most of the time. The form of the memory kernel suggests that the larger the lag time the longer the memory the system retains. This behavior shows that a repeated pattern being formed by increasing the lag time, or in other words, memory of the past contributes to the formation of the present.

In the same year (2010), the behavior of the temperature in the first regime suggests that a subdiffusional process takes place, and an exponent value of 0.806 characterizes the change of the variance (see Fig. 4). The temperature mean–field is inhomogeneous, *C* = 0.103, the high field values dominate the overall process, the Lévy index is 1.152, and the field is non–conservative, thus indicating flux of energy and dissipation, *h* = 0.478. In the second regime, the temperature mean–field is quite homogeneous, *C* = 0.051, the extreme values of the field do not contribute as much as they would in the changes of the field in the first regime, *α* = 1 Cauchy–Lorentz distribution. The temperature mean field remains non–conservative, *h* = 0.224, and the exponent that characterizes the changes of the variance is 0.376. Finally, in the third regime (where the structure function is not shown in Fig. 3) the variance scales with an exponent of 0.305. In all regimes, the temperature changes seem to obey a subdiffusive process with some additional features, when compared to the behavior of the conductivity changes. As temperature expresses the dissipation of energy, its variance in the three different regimes (none of them goes to 0) indicates a continuous flux of energy into the seawater resulting directly in a rise of temperature. The system is far from equilibrium, in agreement with the huge anomalies observed in CTD profiles^7^.

**Figure 2:**
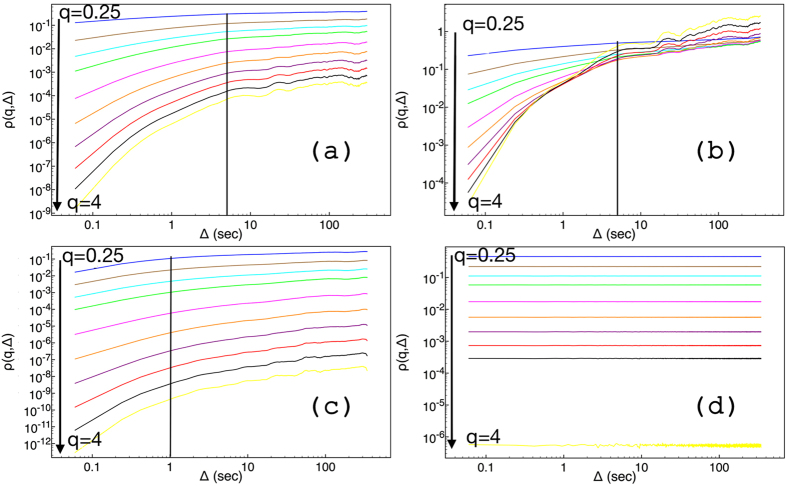
Logarithmic plots of moments of different orders, for *q* = 0.25, 0.5, 0.75, 1, 1.5, 2, 2.5, 3, 3.5, and 4. The upper panel shows the moments for 2010, *E*_*c*_ (**a**) and Θ (**b**). The lower panel shows the same moments for 2011, *E*_*c*_ (**c**) and Θ (**d**). The arrows illustrate the order of the moment.

**Figure 3:**
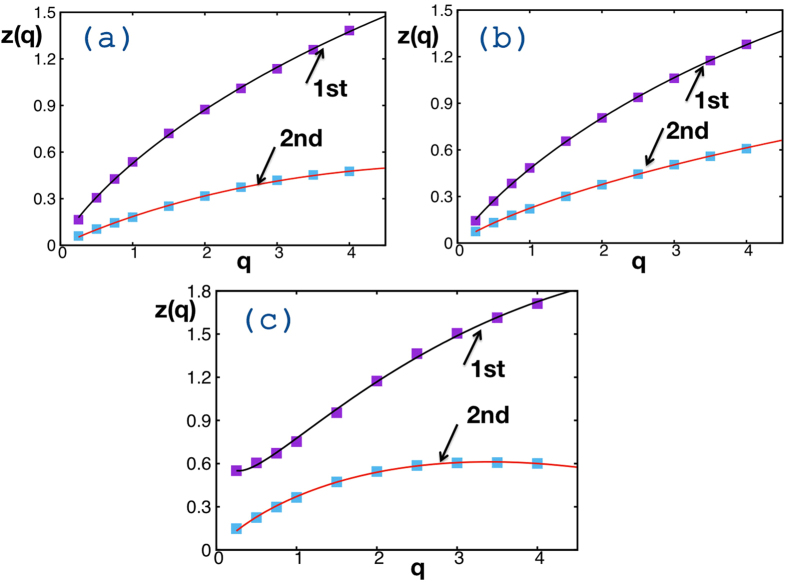
The structure function *z(q*) vs. the *q*–moment order is depicted for 2010 data of *E*_*c*_ (**a**) and Θ (**b**), and for 2011 *E*_*c*_ data (**c**). For 2011 data of Θ, *z(q*) takes null values for every value of *q*. The arrows point to different regimes of Δ as indicated in Fig. 2.

**Figure 4:**
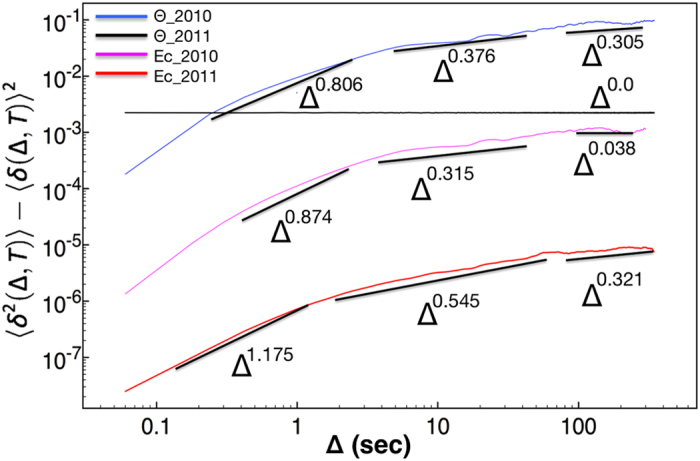
The variance vs. lag time. Top curve is for Θ 2010, middle is for *E*_*c*_ 2010, and bottom is for *E*_*c*_ 2011. Θ 2011 is parallel to the time axis (black line). The variance of each quantity has been fitted by equation (1), *W*(Δ) = *K*_*γ*_Δ^*γ*^, for each regime. The parameters *K*_*γ*_, *γ* are listed in Table 2.

**Figure 5:**
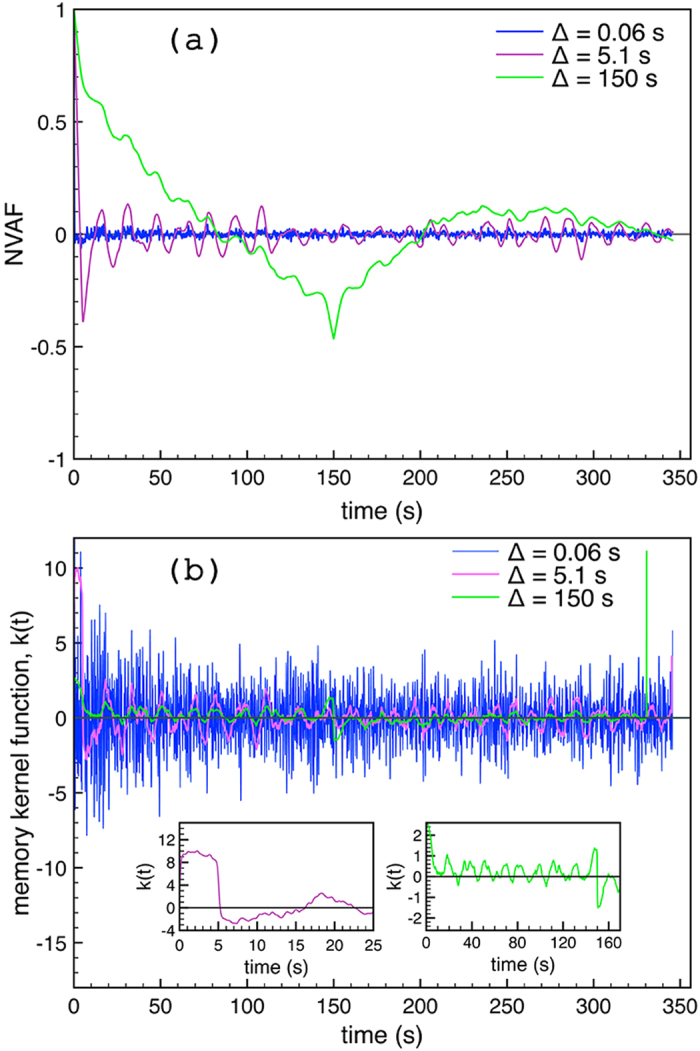
The normalized velocity autocorrelation function (NVAF), *v*^(Δ)^(*t*)/*v*^(Δ)^(0), versus time *t* is illustrated in panel (a). Curves correspond to the minimum lag time 0.06 s (blue), a lag time of 5.1 s (purple), and a lag time of 150 s (green). Each lag time corresponds to a different scaling law in Fig. 4. For the same lag times, *k(t*) functions are illustrated in panel (b). The panel (b) contains two insets. Each inset shows the time dependence of the memory kernel for time moments a bit larger than the lag time for each case; at the left (purple) for lag time of 5.1 s, and at the right (green) for a lag time of 150 s.

Instead, in 2011, temperature, see (Fig. 1d), is practically constant as this is illustrated by both the variance, where *γ* = 0.0, see black horizontal line in Fig. 4, and the GMM where all moments are parallel to horizontal axis (Fig. 2d). If a diffusing particle were under study, this observation would correspond to a particle trapped with its position unchanged, *γ* = 0. Additionally, *h* = 0 and the temperature mean field is conservative, thus no energy flux is expected. Indeed in 2011, measurements repeated over the exact same Kolumbo’s vent field locations showed absence of activity (at least for the time interval reported here). These findings assure that no dissipation exists. Therefore constant Θ at the interface between the two subsystems, seawater and crater subfloor, would be expected. The latter corresponds to a non–equilibrium steady state as the behavior of conductivity suggests for the same period, see discussion below.

Hydrothermal venting has ceased intense activity, retaining a very weak two–way exchange of material between the crater subfloor and the seawater, being in a non–equilibrium state. 2011 *E*_*c*_ data satisfy equation (4) with an additional logarithmic correction term. The *E*_*c*_ mean field is inhomogeneous, *C* = 0.131, non–conservative, *h* = 0.776, its changes are drawn by steps taken from a 1.454 Lévy distribution, and the overall behavior is slightly superdiffusive (Fig. 4). Superdiffusion exhibits persistent behavior that can be explained by a temporal increase of conductivity, likely followed by another increasing step. In the second regime, the mean inhomogeneity of the *E*_*c*_ field is practically unchanged, *C* = 0.134, and the *E*_*c*_ field remains non–conservative, *h* = 0.371, and a 1.243 Lévy index indicates that extreme values play a significant role in the changes of the field. The overall process is subdiffusive, and this still holds even in the third regime of the variance, where a smaller exponent is obtained. This behavior illustrates the fact that in this non–equilibrium steady state, the changes of the conductivity are rapid and constructive over the first regime, and anti–persistent otherwise. No trapping effects are observed, suggesting that memory effects are much weaker than the case of the active field in the formation of repeated patterns. On the other hand, the motion of ions in the immediate surroundings creates a magnetic field, which makes the conductivity mean field non–conservative.

## Summary and Discussion

Kolumbo is considered one of the most dangerous submarine volcano in the Mediterranean^4^. There are clearly different signatures in the way the hydrothermal venting occurs across periods of different activity, influencing the conductivity and temperature in time. Understanding the underlying processes by studying data patterns and trends can be invaluable for assessing the actual risks due to potential explosive unrest.

The analysis of *in situ* recorded data over Kolumbo’s hydrothermal vent field shows for the first time that conductivity behavior may be classified as universal multifractal for both active and inactive data sets. Motion of ions produces magnetic fields that modify conductivity mean field into a non-conservative field. The presence of a non–conservative field affects the process of conductivity change that never attains real equilibrium and cannot show a truly random behavior. This fact holds true even for the inactive period where tiny variations of the conductivity indicate that the system is in a non–equilibrium steady state. In addition, conductivity shows to follow repeated patterns result of memory kernel formation, which becomes much weaker in the case of the rest period. On the other hand, temperature for the inactive periods is a conservative field and no energy dissipation exists. Instead, for the active periods, temperature is also classified as universal multifractal with a continuous flux of energy into the environment.

## Additional Information

**How to cite this article**: Bakalis, E. *et al*. Breathing modes of Kolumbo submarine volcano (Santorini, Greece). *Sci. Rep.* **7**, 46515; doi: 10.1038/srep46515 (2017).

**Publisher's note:** Springer Nature remains neutral with regard to jurisdictional claims in published maps and institutional affiliations.

## Figures and Tables

**Table 1:** 

	*E_c_* (2010)	Θ (2010)	*E_c_* (2010)	Θ (2010)
	0 < *t* ≤ 5		5 < *t* ≤ 150	
*h*	0.528 ± 0.004	0.478 ± 0.002	0.185 ± 0.002	0.224 ± 0.001
*C*	0.133 ± 0.003	0.103 ± 0.004	0.030 ± 0.004	0.051 ± 0.001
*α*	1.0	1.152 ± 0.045	1.619 ± 0.138	1.0
	***E_c_*** **(2011)**	**Θ (2011)**	***E_c_*** **(2011)**	**Θ (2011)**
	**0 < *****t*** **≤ 1**		**1 < *****t*** **≤ 100**	
*h*	0.776 ± 0.011	—	0.371 ± 0.002	—
*C*	0.137 ± 0.031	—	0.134 ± 0.004	—
*α*	1.454 ± 0.238	—	1.243 ± 0.138	—
*d*	0.232 ± 0.021	—	—	—

The values of the parameters *h, C, α*, as well as the value of the logarithmic correction term *d* in equation (4) (*q* ≤ 4).

**Table 2:** Parameters *K*_*γ*_, *γ* used in equation (1), matching the variance of conductivity and temperature in each regime.

	*E*_*c*_ (2010)		
	0 < *t* ≤ 5	5 < *t* ≤ 150	*t* > 150
*K*_*γ*_	(600 ± 7) × 10^−6^	(1500 ± 6) × 10^−6^	(54 ± 2) × 10^−4^
*γ*	0.874 ± 0.009	0.315 ± 0.001	0.038 ± 0.008
	***E***_***c***_ **(2011)**		
	**0 < *****t*** **≤ 1**	**1 < *****t*** **≤ 100**	***t***** > 100**
*K*_*γ*_	(1000 ± 7) × 10^−9^	(150 ± 1) × 10^−8^	(400 ± 5) × 10^−8^
*γ*	1.175 ± 0.016	0.545 ± 0.002	0.321 ± 0.002
	**Θ (2010)**		
	**0 < *****t*** **≤ 5**	**5 < *****t*** **≤ 150 s**	***t***** > 150 s**
*K*_*γ*_	0.049 ± 0.0004	0.088 ± 0.0003	0.110 ± 0.002
*γ*	0.806 ± 0.006	0.376 ± 0.001	0.305 ± 0.003

For Θ 2011 *γ* is null everywhere and is not shown.
